# A human sleep homeostasis phenotype in mice expressing a primate-specific *PER3* variable-number tandem-repeat coding-region polymorphism

**DOI:** 10.1096/fj.13-240135

**Published:** 2014-06

**Authors:** Sibah Hasan, Daan R. van der Veen, Raphaelle Winsky-Sommerer, Alexandra Hogben, Emma E. Laing, Frank Koentgen, Derk-Jan Dijk, Simon N. Archer

**Affiliations:** * Faculty of Health and Medical Sciences, University of Surrey, Guildford, UK; and; † Ozgene Pty. Ltd., Bentley, Western Australia, Australia

**Keywords:** circadian, sleep deprivation, EEG, clock genes

## Abstract

In humans, a primate-specific variable-number tandem-repeat (VNTR) polymorphism (4 or 5 repeats 54 nt in length) in the circadian gene *PER3* is associated with differences in sleep timing and homeostatic responses to sleep loss. We investigated the effects of this polymorphism on circadian rhythmicity and sleep homeostasis by introducing the polymorphism into mice and assessing circadian and sleep parameters at baseline and during and after 12 h of sleep deprivation (SD). Microarray analysis was used to measure hypothalamic and cortical gene expression. Circadian behavior and sleep were normal at baseline. The response to SD of 2 electrophysiological markers of sleep homeostasis, electroencephalography (EEG) θ power during wakefulness and δ power during sleep, were greater in the *Per3*^5/5^ mice. During recovery, the *Per3*^5/5^ mice fully compensated for the SD-induced deficit in δ power, but the *Per3*^4/4^ and wild-type mice did not. Sleep homeostasis-related transcripts (*e.g.*, *Homer1*, *Ptgs2*, and *Kcna2*) were differentially expressed between the humanized mice, but circadian clock genes were not. These data are in accordance with the hypothesis derived from human data that the *PER3* VNTR polymorphism modifies the sleep homeostatic response without significantly influencing circadian parameters.—Hasan, S., van der Veen, D. R., Winsky-Sommerer, R., Hogben, A., Laing, E. E., Koentgen, F., Dijk, D.-J., Archer, S. N. A human sleep homeostasis phenotype in mice expressing a primate-specific *PER3* variable-number tandem-repeat coding-region polymorphism.

There are individual differences in sleep timing and duration and responses to sleep loss, but the underlying mechanisms remain poorly understood (1, 2). Sleep-wake cycles are determined by the interaction of circadian timing and sleep homeostasis (3, 4), and some of the molecular mechanisms of both processes have been identified.

Cellular circadian rhythms are generated by a transcriptional–translational feedback oscillator consisting of the positive regulators CLOCK and BMAL1 and the negative regulators Period (PER) and Cryptochrome (CRY), which inhibit expression (5). Alterations in these genes are associated with the timing of sleep-wake activity and sleep homeostasis (6). In particular, during non-rapid eye movement sleep (NREMS), electroencephalography (EEG)-measured δ power, an established marker of sleep homeostasis, is affected by clock genes (7, 8). Sleep duration is associated with single-nucleotide polymorphisms (SNPs) in *CLOCK* (9), and sleep-timing preference and delayed sleep-phase disorder (DSPD) are associated with polymorphisms within *PER2*, *PER3*, and *CLOCK*, although the results have not always been replicated (2). Most human association studies have not shown whether polymorphisms affect the circadian or homeostatic aspect of sleep regulation, and many show no direct link between specific polymorphisms and causative mechanisms (2).

Although *Per3* was originally described as a clock gene and data show that it interacts with other clock elements (10), its redundant role within the circadian clock is far from clear (11, 12). Functional knockout (KO) of PER3 in mice has a minimal effect on the central circadian clock (11–14), but it alters sleep homeostasis (15). A primate-specific variable-number tandem-repeat (VNTR) polymorphism (54 bp, repeated 4 or 5 times) in human *PER3* associates with diurnal preference (16–19), DSPD (16, 17), sleep–wake timing (19), sleep homeostasis (20, 21), and cognitive vulnerability to sleep loss (20, 22–24). The *PER3* VNTR is not associated with major changes in either circadian phase (20, 25, 26) or circadian period, assessed *in vivo* or *in vitro* (27). We therefore hypothesized that the *PER3* VNTR affects sleep homeostasis, rather than circadian rhythmicity (28).

In this study, we sought to investigate whether the above-mentioned associations between the *PER3* VNTR polymorphism and human sleep phenotypes reflect a direct causative effect of the polymorphism on homeostatic, but not on circadian, sleep regulation. We created 2 separate transgenic mouse lines expressing the human 4- and 5-repeat of the *PER3* VNTR and assessed behavioral circadian rhythmicity and EEG-assessed aspects of sleep homeostasis. Sleep deprivation (SD) is known to affect cortical clock gene expression in rodents (29), and the hypothalamus contains both the circadian clock in the suprachiasmatic nuclei (SCNs) and the ventrolateral preoptic (VLPO) nucleus, which regulate sleep–wakefulness (30). We therefore assessed the effects of the VNTR on sleep-associated gene expression in the cortex and hypothalamus in these humanized mice.

## MATERIALS AND METHODS

### Generation of C57BL/6 transgenic mice

**Figure 1.**
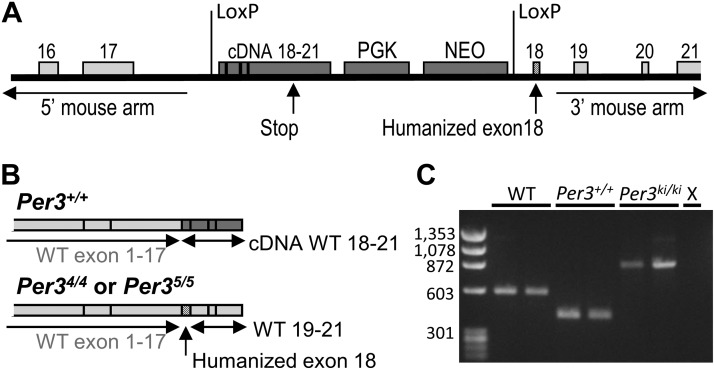
*A*) Schematic representation of the humanized *Per3* targeted alleles. A cDNA insert containing mouse exons 18–21 was inserted between 2 LoxP excision sites in mouse intron 17, replacing exon 18. After the second LoxP excision site, the human exon 18 (coding for either the 4- or 5-repeat VNTR allele) replaced mouse exon 18, followed by mouse intron 18 and the subsequent WT mouse homology arm sequence. *B*) Top: transgenic individuals express mRNA containing WT mouse exons 1–17, followed by the cDNA-coded WT exons 18–21. Bottom: after Cre-Lox recombination, humanized mice also express WT mouse exons 1–17, now followed by human exon 18 and WT mouse exons 19–21. *C*) Representative PCR products for genotyping of WT, *Per3*^+/+^, and humanized mice. *Per3*^*KI/KI*^, humanized KI; X, negative control.

Targeting vectors were created by PCR from C57BL/6 genomic DNA using the plasmid FLSniper as the vector backbone (Ozgene Pty. Ltd., Bentley, WA, Australia). Targeting vectors were designed to conditionally replace mouse exon 18 with human exon 18, coding for either the 4- or 5-repeat VNTR (**Fig. 1*A***). The targeting construct contained a cDNA sequence of wild-type (WT) mouse exons 18–21, with a downstream phosphoglycerine kinase (PGK) promoter driving a neomycin (Neo) selection marker. The sequence was flanked by locus of X over P1 (LoxP) excision sites and followed by the human *PER3* exon 18, containing either the 4- or 5-repeat allele of the VNTR. For the known SNP T3110C within the VNTR (31), we chose to express the more common allele. Gene targeting was performed in the embryonic stem (ES) cell line Bruce4, which was derived from a C57BL/6-Thy1.1 congenic strain (32). Chimeras were crossed to C57BL/6J. The genetic background of the resulting mice is C57BL/6, with minor polymorphic contributions from the ES cells (33). The vector was introduced into the ES cells by electroporation, and successful homologous recombination was confirmed by screening for the antibiotic-resistant ES cells. After confirmation of integration of the targeting vector by Southern blot, positive ES cells were expanded and injected into blastocysts, which were then implanted in pseudopregnant females (C57BL/6), and chimeras were bred to obtain germline transmission.

ES-derived coat-color offspring were genotyped with Southern blot analysis and used as founders for the breeding colony. These conditional (*Per3*^+/+^) mice expressed a WT protein coded by the WT gene (exons 1–17) and transgenic cDNA WT sequence (exons 18–21; see **Fig. 1*B***). Design and construction of the targeting vectors and creation of transgenic offspring were performed by Ozgene.

By applying tyrosine recombinase/locus of X (Cre-Lox) recombination, humanized knock-in (KI) and WT mice were created by crossing homozygous C57BL/6 transgenic mice with mice on the same background that expressed *Cre* ubiquitously under the control of the PGK promotor at the ROSA26 locus (Ozgene). The F1 offspring of this cross were heterozygous for both *Cre* and the humanized *Per3* construct. These offspring were used in selective breeding to create mice homozygous for the KI construct and not expressing *Cre* [*Per3* homozygous for the 4-repeat or 5-repeat allele (*Per3*^4/4^ and *Per3*^5/5^, respectively) mice] and mice homozygous for the WT mouse *Per3* gene and not expressing *Cre*. These mice were used to establish the lines used in the experiments. Note that the KI and WT mice were derived from the same conditional transgenic mice after selective crossing with the *Cre* mice and were genetically identical, except for the presence or absence of the VNTR.

Two PCR primer sets were used for genotyping. Set 1 was directed at the homology arms (forward: 5′-AGCCGGGGTCAGAGCACAGTAGTT-3′; reverse: 5′-ATCCATGAGCCCAAGCAAATCCT-3′), and set 2 was directed at PGK-NEO inserts (forward: 5′-GTCTGCCGCGCTGTTCTCCTCTTC-3′; reverse: 5′-CTTCGCCCAATAGCAGCCAGTCC-3′). The combination of these primers resulted in PCR products of 583 bp (set 1) in WT mice, 418 bp (set 2) in conditional *Per3*^+/+^ mice, and 886 bp (set 1) in *Per3*^4/4^ and *Per3*^5/5^ mice (Fig. 1*C*). Primer set 2 was also used to identify *Cre*-expressing mice, with a PCR product of 354 bp. Direct sequencing of *Per3* transcript copy DNA showed that these mice expressed the expected KI constructs.

### Housing conditions

Humanized *Per3*^4/4^ and *Per3*^5/5^ mice and their WT controls were bred in-house from homozygous breeding pairs. During experimentation, the mice were individually housed in light-tight, sound-attenuated cabinets, with food and water provided *ad libitum*. The mice were maintained on a 12-h light-dark (LD) cycle, with a light intensity of 800 ± 13.4 mW/m^2^ at the level of the cage bottom during the light period, and were kept at a controlled ambient temperature (20–22°C) and relative humidity (55±10%). Nesting material (bedding) was provided to aid thermoregulation. All experimental procedures received a favorable opinion by the University of Surrey Animal Ethics Committee and were carried out under a U.K. Home Office License and in accordance with the Declaration of Helsinki.

### Circadian period protocol

WT and transgenic C57BL/6 mice (*n*=16/genotype, 7 females and 9 males; age 64 ± 2 d; mean±sem) were individually housed with a 12-h LD cycle. After 14 d, the mice were exposed to four 14-d episodes of constant light of increasing intensity (0, 33, 171, and 865 mW/m^2^) and a final 14-d episode of constant darkness. The blue-enriched LED light spectrum did not vary between intensities. Running-wheel activity (1 min resolution) was recorded throughout the experiment (ClockLab; Actimetrics, Wilmette, IL, USA). During the last 10 d for each light condition, the length of the free-running circadian behavioral period was calculated by periodogram analysis (34). Free-running periods were compared by using PROC MIXED in SAS 9.2 (SAS Institute, Cary, NC, USA) with the factors genotype, sex, and light intensity. Representative activity profiles were double plotted for each genotype as qualitative actograms, and free-running periods are indicated as group means ± sem.

### Duration and temporal pattern of EEG-assessed vigilance states (VSs)

A telemetric transmitter (volume, 1.9 cm^3^; total weight, 3.4 g; TL11M2-F20-EET; DSI, St. Paul, MN, USA) connected to electrodes for continuous EEG and electromyography (EMG) recordings was implanted in 8 adult male C57BL/6 mice per genotype (*Per3*^4/4^, *Per3*^5/5^, and WT; *N*=24, 78.7±0.7 d old; mean±sem). Body weights were not similar between the genotypes [WT, 29.0±1.3 g; *Per3*^4/4^, 26.5±1.8 g; *Per3*^5/5^, 29.5±2.0 g (avg.±sd); *P*<0.005; PROC MIXED]. The *Per3*^4/4^ mice weighed less than the WT (*P*<0.05) and *Per3*^5/5^ mice (*P*<0.01; LSMEANS after Tukey adjustment). In mice under anesthesia (isoflurane induction 3.6%, maintenance 1–2%), 2 stainless-steel EEG electrodes (length of screw shaft, 2.4 mm; outer diameter of screw thread, 1.19 mm) were implanted epidurally over the right frontal and parietal cortices (35) and connected to the telemetry transmitter *via* medical-grade stainless-steel wires (surrounded by silicone tubing). The EEG electrodes and connections to the subcutaneous wiring were covered with dental cement (GC Fuji Plus; GC United Kingdom Ltd., Newport Pagnell, UK). Two EMG stainless-steel leads were inserted into the neck muscle ∼5 mm apart and sutured in place. The telemetry transmitter was placed in a subcutaneous pocket and positioned along the left dorsal flank of the mouse. Analgesia was administered by subcutaneous injection at the onset of surgery (Vetergesic, 30 μg/kg). After surgery, the animals were allowed to recover for 11 ± 1 d (mean±sd), and baseline (BSL) data collection was begun when the mice were 89.6 ± 0.8 wk of age (mean±sem).

The telemetric transmitters were activated 2 d before the BSL day, and EEG/EMG signals were then recorded continuously for 72 h (Dataquest ART; DSI). The EEG and EMG signals were modulated with a high-pass (3 dB, 1.0 Hz) and a low-pass antialiasing (48 Hz) analog filter. The data analyzed consisted of continuous 72-h recordings, including a 24-h BSL period starting at light onset [zeitgeber time (ZT) 0], followed by a 12-h period of SD in light (SD-L) and a subsequent 36-h period of sleep recovery (Rec).

SD was carried out starting at light onset (ZT 0) for 12 consecutive hours, by the simultaneous presence of at least 2 (ZT 0–9) or 3 researchers (ZT 9–12) for each group of 8 mice (of mixed genotypes). SD was performed by sequential introduction of different novel objects into the cage when the animal appeared to become drowsy (up to 14 in total, consisting of nesting material, blue tissue, open or closed cardboard rolls of different sizes, pieces of wood, chemiwood prisms, plastic tubes of different sizes, tube lids, and Petri dishes). If necessary, additional arousal was achieved by disturbing the bedding and/or the nest. The cages were exchanged twice (at ZT 6 and 9) for fresh ones, to provide additional stimulation. Researchers were blinded to genotype during SD, and the objects were introduced into the cages in the same order.

The telemetric EEG and EMG data were transmitted at 455 kHz to an RPC-1 receiver (DSI) and sampled at 250 Hz. VSs for consecutive 4 s epochs were classified by visual inspection of the EEG and EMG signals, according to standard criteria (35), as follows: wakefulness (high and variable EMG activity and a low-amplitude EEG signal); NREMS (high EEG amplitude, dominated by slow waves and low EMG); and rapid eye movement sleep (REMS; low EEG amplitude, θ oscillations of 5–9 Hz, and loss of EMG muscle tone).

The time spent in each VS was expressed as a percentage of total recording time over 2 time intervals (*i.e*., 2 or 12 h). REMS was also expressed as a percentage of total sleep time (TST). The effects of SD on Rec NREMS and REMS were assessed as accumulated differences from the BSL condition for consecutive 1-h intervals starting at light onset (ZT 0).

### EEG power density

EEG power spectra were computed for consecutive 4-s epochs by a fast Fourier transform (FFT; frequency range, 0.98–47.85 Hz; resolution, 0.244 Hz; Hanning window function). Only artifact-free epochs (97%) were included in the EEG spectral analyses. EEG power spectra were determined for NREMS, REMS, and wakefulness during the 12-h BSL light (BSL-L) period. EEG power spectra are expressed as a percentage of total EEG power (frequency range: 0.98–47.85 Hz; resolution: 0.24 Hz; graphed frequency range: 0.98–31.98 Hz).

The changes in the waking EEG spectra during 12 h SD were determined by expressing EEG spectra individually as a percentage of the wakefulness spectra in the 12-h BSL-L period (range: 0.98–47.85 Hz; resolution: 0.24 Hz). The time course of EEG spectra changes during forced wakefulness in SD was assessed by dividing this period into 36 time intervals, in each of which an equal number of 4-s awakening epochs were contributed by the individual mice. The θ peak frequency (TPF) in REMS was determined by recording the frequency at which EEG power density was at its maximum.

To further quantify the changes in the awake EEG spectra, the following frequency ranges were defined: upper θ, 8.5–11 Hz; β-2, 20–35 Hz; and γ-1, 35–48 Hz. Mean EEG power in these frequency bands was expressed as a percentage of the mean EEG power reached in that specific band during wakefulness in the BSL-L period.

EEG δ power during NREMS was computed by averaging the EEG power in the frequencies ranging from 0.98 to 3.91 Hz. Mean values were calculated by dividing the 12-h periods into 12 (BSL-L); 5 [BSL-dark (BSL-D)]; 9 [12-h period following SD: first Rec dark (Rec-D1)]; 11 [Rec light (Rec-L)]; or 6 [second Rec dark (Rec-D2)] intervals to which an equal number of NREMS epochs contributed within each mouse. Mean values were also individually normalized to the mean δ power in NREMS in the last 4 h of the BSL-L period (*i.e*., the lowest mean level reached during BSL). The δ power rebound during the first interval after SD was calculated as a percentage of δ power within the interval immediately preceding SD. Accumulated differences (from the BSL condition) of δ power during NREMS are represented as hourly values of δ energy (*i.e*., summed δ power over all epochs) and expressed as a percentage of the total δ energy during the 24 h of the BSL day.

### Statistics for sleep analyses

Data were analyzed with SAS v9.2 (SAS Institute). For the EEG power spectra (per VS during BSL-L), the spectral changes in the awake EEG (during SD-L), and the time courses of accumulated differences in NREMS, REMS, and slow-wave energy (SWE) during SD and the 36-h Rec period after SD, nonparametric analyses were used with PROC NPAR1WAY (Kruskal-Wallis test), followed by the Student-Newman-Keuls (SNK) *post hoc* analysis. The TPF data between the genotypes were compared by using PROC TTEST. PROC MIXED was also used to compute intraclass correlation coefficients (ICCs). ICCs were computed with their 95% confidence intervals (CIs). In our study, the ICC indicates the ratio of the between-genotypes (*Per3*^4/4^ and *Per3*^5/5^ only) variance to the total variance (within-genotype variance + between-genotypes variance). ICCs can have any value between 0 and 1; higher values are indicative of stability of observations within a genotype (or genotype effect). At BSL, VS data (per 2 and 12 h), and time courses of δ, upper-θ, β-1, and γ-1 power were analyzed by using PROC MIXED for analysis of variance (ANOVA; factor genotype and interaction genotype × time; additionally, factor SD and interaction SD × time for VS per 12 h only), followed by LSMEANS *post hoc* multiple pair-wise comparisons. PROC MIXED was also used to assess genotype effects on the immediate δ power rebound (using the log_10_ values).

### Microarray gene expression analysis

To investigate the effects of the insertion of the VNTR transgene on gene expression in the hypothalamus and cortex of the *Per3* KI mice, we used the same 12-h SD protocol. After 14 d of entrainment to a 12-h LD cycle, SD was carried out as described for the sleep EEG experiment for each group of 8 mice [of mixed genotypes; *n*=8/genotype (*Per3*^4/4^, *Per3*^5/5^ and WT); age 102 ± 2.4 d; mean±sem]. At the end of the SD period, whole brains were collected under red light and stored immediately at −80°C in RNAlater (Qiagen, Hilden, Germany) until RNA extraction from the hypothalamus and cortex tissue with the RNeasy kit (Qiagen). Total RNA (200 ng) was used to synthesize the labeled cRNA (Quick Amp; Qiagen), which was hybridized to Whole-Mouse Genome 4×44K slides (design ID 014868; Agilent Technologies, Santa Clara, CA, USA). Array features were extracted with Agilent Feature Extraction software.

We also measured hypothalamic gene expression in a protocol where behavioral activity is suppressed by light (13). After 10 d in the 12-h LD cycle, mice (male C57BL/6 mice: *n* = 8/genotype; age 75 ± 3 d, mean±sem) received 17 cycles of 3.5 h light (170±2.3 mW/m^2^):3.5 h dark, starting at original light onset. Brains were collected in the 17th light cycle (where the largest behavioral difference between WT and *Per3* KO mice had been observed; ref. 13) and stored at −80°C in RNAlater (Qiagen). RNA extracted from the hypothalamus microarrays was hybridized with labeled cRNA as described above. Samples of 1 *Per3*^5/5^ and 1 WT mouse were excluded from further analyses because of the poor quality of the arrays, based on the output of the Agilent feature extraction.

Expression levels were analyzed with GeneSpring 12 (Agilent). The samples were normalized by percentile shift to the 75th percentile. The probes were classified as differentially expressed with avalue of α = 0.05 in a 1-way ANOVA and unpaired *t* test (asymptotic *P* value computation with Benjamini-Hochberg correction for multiple testing) and/or a probability of false prediction (PFP) < 0.015 in a rank product analysis (36).

Gene Ontology (GO) analysis of the differentially expressed probes was conducted in WebGestalt (37) using the mouse 4×44K (ver. 1; Agilent) as the background distribution. All microarray data have been deposited in the U.S. National Center for Biotechnology Information (Bethesda, MD, USA) Gene Expression Omnibus (GEO) database (GEO accession number GSE55255; http://www.ncbi.nlm.nih.gov/geo/).

## RESULTS

### Circadian period in constant dark and constant light

**Figure 2.**
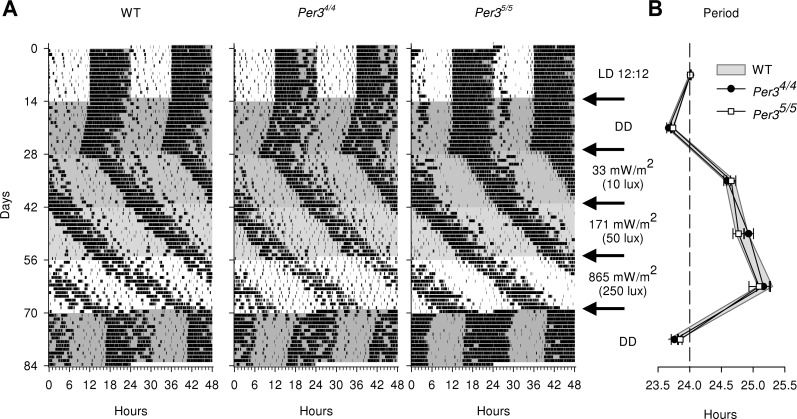
Circadian period in constant dark and constant light. *A*) Examples of individual activity records (per 24 h, double plotted) for WT, *Per3*^4/4^, and *Per3*^5/5^ mice. Activity is represented by black bars. Successive light conditions (white, light; gray, continuous light or dark) over the 84-d period are indicated at right. Mice were first entrained to a 12-h LD cycle and then were exposed to a period of constant darkness (DD), followed by 3 successive conditions of increasing levels of constant light, before returning again to DD (arrows). *B*) For each light condition, the circadian free-running period was calculated from periodogram analysis over the last 10 d of that condition and is shown as average ± sem per genotype per light condition.

Representative examples of individual records of behavioral activity for each genotype are shown in **Fig. 2*A***. During the 12-h LD cycle, behavioral rest-activity cycles were entrained to the 24-h period in all genotypes. Free-running behavioral activity rhythms were observed in all constant-light and constant-dark conditions. As expected (38), the free-running circadian period lengthened with increasing light intensities (main effect of light, *P*<0.0001), and no significant main effect of genotype or interaction between genotype and light was observed (all genotype and light∗genotype, *P*>0.05; Fig. 2*B*). The females exhibited a larger increase in period length than did the males in response to the dimmest and brightest light conditions (light∗sex, *P*<0.005, LSMEANS contrast *P*<0.0005 and 0.0001 for 33 and 865 mW/m^2^, respectively). This sex difference was observed in all mice and was not affected by genotype (light∗sex∗genotype, *P* > 0.05).

### VSs and EEG power spectra at BSL

During the BSL day, the 12-h averages of time spent in wakefulness or in NREMS or REMS were similar in the *Per3*^4/4^, *Per3*^5/5^, and WT mice (**Table 1**).

**Table 1.** VSs and δ power during 12 h NREMS

Parameter	BSL-L	BSL-D	sd-L	Rec-D1	*P vs.* BSL-D	Rec-L	*P vs.* BSL-L	Rec-D2	*P vs.* BSL-D
Awake (%)									
WT	38.2 ± 1.2	77.9 ± 2.4	98.88 ± 0.09	58.0 ± 4.2	<0.0001	40.7 ± 1.3	<0.05	73.3 ± 3.0	<0.01
*Per3*^5/5^	38.7 ± 1.1	77.0 ± 1.6	99.20 ± 0.09	57.6 ± 1.5	<0.0001	41.2 ± 1.4	<0.05	71.6 ± 1.2	<0.001
*Per3*^5/5^	40.9 ± 1.6	76.3 ± 1.8	99.16 ± 0.09	53.4 ± 1.8	<0.0001	42.3 ± 1.8		68.5 ± 1.8	<0.0001
NREMS (%)									
WT	46.8 ± 1.6	19.3 ± 2.1	1.13 ± 0.26	32.7 ± 3.3	<0.0001	44.5 ± 1.7		22.9 ± 2.6	<0.05
*Per3*^5/5^	46.1 ± 1.4	20.1 ± 1.5	0.80 ± 0.09	32.3 ± 1.7	<0.0001	42.1 ± 1.9	<0.05	23.9 ± 1.3	<0.01
*Per3*^5/5^	44.6 ± 1.2	20.6 ± 1.6	0.84 ± 0.09	36.4 ± 1.8	<0.0001	41.9 ± 1.5		26.4 ± 1.6	<0.001
REMS (%)									
WT	15.0 ± 1.1	2.8 ± 0.4	0.00 ± 0.00	9.4 ± 1.2	<0.0001	14.8 ± 1.2		3.9 ± 0.5	
*Per3*^5/5^	15.2 ± 0.9	2.8 ± 0.2	0.00 ± 0.00	10.1 ± 0.8	<0.0001	16.8 ± 1.4		4.6 ± 0.3	<0.001
*Per3*^5/5^	14.4 ± 1.2	3.1 ± 0.3	0.00 ± 0.00	10.2 ± 0.8	<0.0001	15.8 ± 1.6		5.1 ± 0.4	<0.0001
REMS/TST (%)									
WT	24.3 ± 1.8	12.3 ± 1.1	NA	21.9 ± 1.3	<0.0001	25.0 ± 2.1		14.4 ± 1.2	
*Per3*^5/5^	24.9 ± 1.5	12.4 ± 0.6	NA	24.0 ± 2.3	<0.0001	28.6 ± 2.3	<0.05	16.3 ± 1.3	<0.05
*Per3*^5/5^	24.3 ± 1.6	12.8 ± 0.8	NA	22.0 ± 1.7	<0.0001	27.2 ± 2.4		16.2 ± 0.9	<0.05
δ power (%)									
WT	100 (ref.)	142.6 ± 5.8	[304.7 ± 15.0]	136.8 ± 8.0		97.3 ± 4.0		152.1 ± 9.3	
*Per3*^5/5^	100 (ref.)	148.8 ± 5.0	[328.1 ± 10.2]	136.4 ± 5.8		95.4 ± 4.5		145.4 ± 8.7	
*Per3*^5/5^	100 (ref.)	146.1 ± 7.6	[364.0 ± 20.9]	155.6 ± 9.6		110.6 ± 7.2		165.1 ± 10.9	

Values represent mean ± sem percentage of time awake, NREMS, and REMS (percentage of total recording time), REMS/TST (percentage TST) and δ power (percentage of BSL-L δ) calculated per 12-h period. *P* < 0.05, *P* < 0.01, *P* < 0.001, and *P* < 0.0001 represent increase or decrease compared to corresponding BSL (PROC MIXED followed by LSMEANS). NA, not applicable.

**Figure 3.**
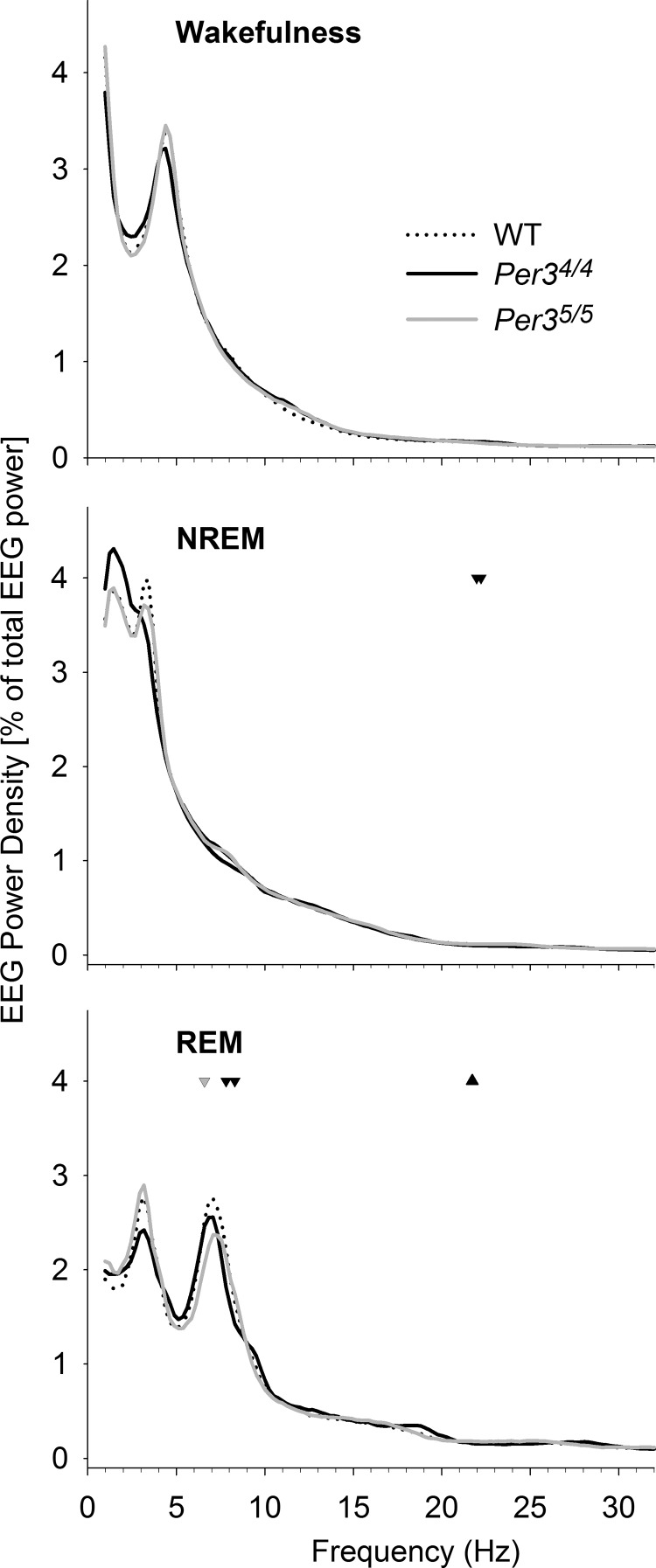
EEG spectral profiles of NREMS, REMS, and wakefulness during the BSL-L period in WT, *Per3*^4/4^, and *Per3*^5/5^ mice. Means (*n*=8/genotype) of the EEG spectra were normalized to total EEG power for all frequencies within corresponding sleep states. Genotype effects are indicated as follows: inverted black triangle, *Per3*^4/4^ lower than both WT and *Per3*^5/5^; gray triangle, *Per3*^5/5^ higher than *Per3*^4/4^; inverted gray triangle, *Per3*^5/5^ lower than both WT and *Per3*^4/4^. All *P* < 0.05 (PROC NPAR1WAY followed by SNK *post hoc*).

The EEG power spectra (in each VS) during the 12-h BSL-L period in the WT, *Per3*^4/4^, and *Per3*^5/5^ mice differed only in narrow frequency ranges (PROC NPAR1WAY, *P*<0.05; **Fig. 3**). During NREMS, the *Per3*^4/4^ mice had lower power in 21.97–22.22 Hz than the *Per3*^5/5^ and WT mice (SNK, *P*<0.05). During REMS, the *Per3*^5/5^ mice showed lower power at 6.59 Hz than the WT and *Per3*^4/4^ mice (SNK, *P*<0.05), as well as higher power at 21.73 Hz compared to the *Per3*^4/4^ mice (SNK, *P*<0.05). In addition, the *Per3*^4/4^ mice had decreased power at 7.81 and 8.30 Hz compared to the WT and *Per3*^5/5^ mice (SNK, *P*<0.05).

To confirm the genotype effect in the θ range of the REM spectrum, we computed the ICCs for each frequency bin by calculating the ratio of the between-genotype variance (*Per3*^4/4^ and *Per3*^5/5^ only) to the total variance (within-genotype variance + between-genotypes variance). In accordance with the observed genotype effect, the highest ICC (0.48) was obtained for the 8.30-Hz bin, indicating stability of power density in the upper-θ range within the genotypes.

### Behavior and EEG-assessed wakefulness during SD

**Figure 4.**
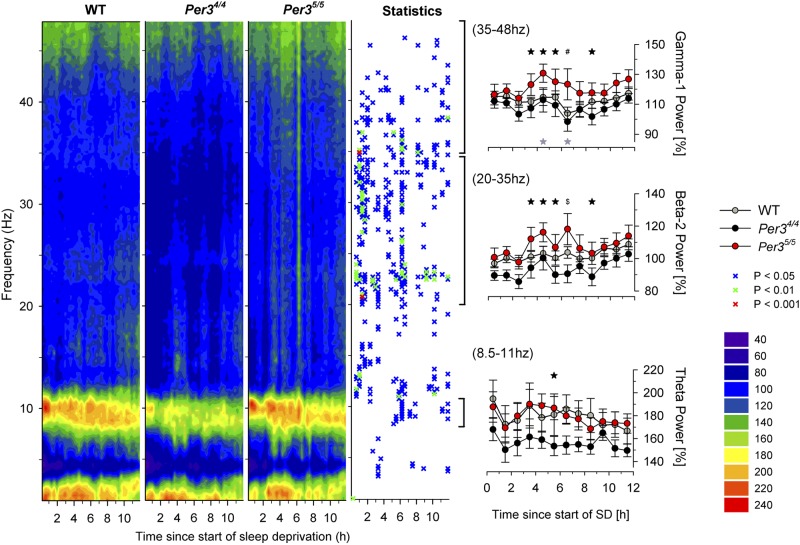
Time course of EEG spectral profiles during the 12-h SD period. Left panel: mean EEG spectra within 36 time intervals were expressed as a percentage of the mean spectral profile for awake EEG in the BSL-L period for each genotype separately. Changes from BSL are plotted as a heat map (key at right): dark blue values represent reduced EEG power compared to BSL; light blue values are similar to BSL (100%); warm colors (green to red) represent increased EEG power compared to BSL. Statistics panel indicates overall genotype effects (PROC NPAR1WAY, *P*<0.05) over time in the full frequency range; symbols (key at right) represent significant genotype effects. Right panels: means ± sem (*n*=8/genotype) of EEG power in the upper-θ (bottom panel), β-2 (middle panel), and γ-1 (top panel) ranges were calculated hourly and expressed as a percentage of the mean EEG power in that range during spontaneous wakefulness in the BSL-L period. Hourly intervals with significant genotype effect. Black symbols indicate significant differences between *Per3*^5/5^ and *Per3*^4/4^; gray symbols indicate significant differences in the β-1 range between *Per3*^5/5^ and WT (PROC MIXED followed by LSMEANS *post hoc*). *P < 0.05, ^#^*P* < 0.01, ^$^*P* < 0.001.

To evaluate and compare the effects of SD on awake EEG in the WT, *Per3*^4/4^, and *Per3*^5/5^ genotypes, EEG spectra were computed over a frequency range of 1 to 48 Hz during the 12 h of SD. In all genotypes, the forced wakefulness during SD was characterized by an immediate and lasting increase (1.4- to 2.4-fold) in the upper-θ-range activity (8.5–11 Hz range), compared to BSL wakefulness during light (power heat map in **Fig. 4**). This θ-range increase was more pronounced in the *Per3*^5/5^ and WT (with an increase up to 2.4-fold) than in the *Per3*^4/4^ mice. Frequencies above θ (>11 Hz) also showed genotype effects (Fig. 4, statistics panel).

To further characterize the effects of SD on awake EEG, we calculated the EEG power per hour in the upper-θ (8.5–11 Hz), β-2 (20–35 Hz), and γ-1 (35–48 Hz) frequency bands. In the upper-θ range, the *Per3*^5/5^ mice showed a significantly higher power during 5–6 h after SD onset compared to *Per3*^4/4^ mice (PROC MIXED *P*<0.05, LSMEANS *P*<0.05). Similarly, in the β-2 and γ-1 range, *Per3*^5/5^ mice showed increased power (compared to *Per3*^4/4^ mice) during the middle of the SD period (PROC MIXED *P*<0.05; for *post hoc* details, see Fig. 4, right panels).

### Rec sleep after SD

During the Rec-D1 period after the 12 h SD, we observed a reduced time spent awake and an increased time spent in NREMS and REMS in all 3 genotypes, compared to that in the BSL-D period (Table 1). Compared to the BSL-L period, wakefulness increased in the WT and *Per3*^4/4^ mice during the 12 h Rec-L period. The *Per3*^4/4^ mice showed an increase in REMS and TST and a decrease in NREMS during the Rec period, compared to BSL (Table 1). During the 12 h Rec-D2 period, wakefulness remained significantly reduced in all 3 genotypes (compared to BSL-D). NREMS remained increased in all 3 genotypes, and NREMS remained increased in the 2 KI genotypes only, compared to the BSL-D period (Table 1).

**Figure 5.**
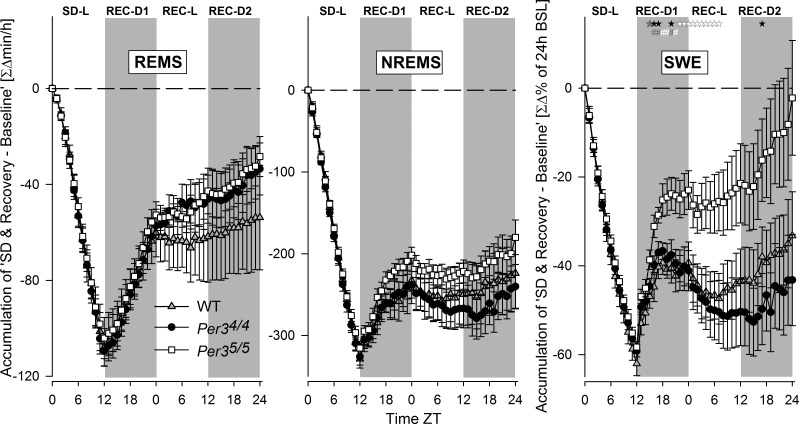
Hourly time course of accumulated differences in REMS time, NREMS time, and SWE between BSL conditions during SD and the 36 h Rec period after SD. Accumulated values (means± sem; *n*=8/genotype) are expressed relative to BSL conditions (black horizontal dashed line) for each genotype (gray triangle, WT; black circle, *Per3*^4/4^; white square, *Per3*^5/5^). Genotype effects are indicated for the hourly intervals for the comparison of SWE accumulation between the 3 genotypes: black symbols indicate significance for *Per3*^5/5^ higher than *Per3*^4/4^; gray symbols for *Per3*^5/5^ higher than WT; white symbols for *Per3*^5/5^ higher than both *Per3*^4/4^ and WT (PROC NPAR1WAY per interval followed by SNK *post hoc*). *P < 0.05, ^#^*P* < 0.01.

To further characterize the effects of SD, we computed the accumulated hourly differences in REMS and NREMS and SWE (δ power in NREMS) between the mean BSL and SD values and the subsequent 36 h Rec period (**Fig. 5**). Negative slopes indicate a loss, null slopes indicate no loss or gain, and positive slopes indicate a gain in sleep time or sleep intensity, relative to BSL. Because each interval is an accumulation of all preceding intervals, statistical analyses were performed only on data from the last 1-h interval. No genotype differences were found in REMS and NREMS times. By contrast, a genotype effect was found for SWE (PROC NPAR1WAY, *P*<0.05), where the *Per3*^5/5^ mice showed a significantly higher SWE accumulation than the *Per3*^4/4^ and WT mice from h 3 of sleep Rec (SNK, *P*<0.05 and *P*<0.01; Fig. 5). In addition, only the *Per3*^5/5^ mice fully compensated for the level of sleep deficit by returning to BSL, whereas, in the *Per3*^4/4^ and WT mice, accumulated levels remained significantly lower than BSL (paired *t* tests: *Per3*^4/4^, *P*<0.005; WT, *P*<0.05; Fig. 5).

When the δ power in NREMS of the first time point of Rec sleep after SD was expressed as a percentage of the δ power during the time point preceding the SD, the *Per3*^5/5^ mice showed a trend for higher levels of δ power than the *Per3*^4/4^ mice (198.15±14.1 *vs.* 168.46±9.01%; PROC TTEST, *P*=0.095).

**Figure 6.**
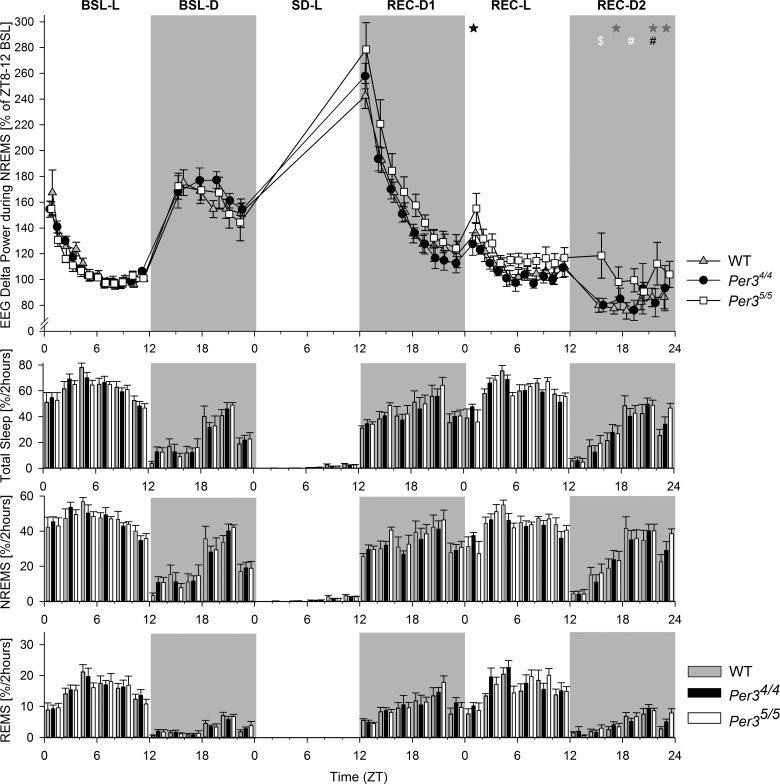
Time courses of EEG δ power, TST, NREMS, and REMS across the entire 72-h period. Top panels: time distribution of EEG δ power during NREMS. EEG δ power (means± sem; *n*=8/genotype) is expressed relative to the last 4 h of the BSL-L period. There was an overall effect of genotype (PROC MIXED LSMEANS, *P*<0.05), and specific contrasts were observed between the 3 genotypes. Black symbols indicate *Per3*^5/5^ higher than *Per3*^4/4^; gray symbols, *Per3*^5/5^ higher than WT; white symbols, *Per3*^5/5^ higher than both *Per3*^4/4^ and WT. *P < 0.05, ^#^*P* < 0.005, and ^$^*P* < 0.0005. Bottom panels: time course of 2-h binned values (means±sem) of total sleep, NREMS, and REMS for the BSL and Rec periods, expressed as percentage of recording time per 2 h. There were no significant genotype effects observed in these measures (PROC MIXED, *P*>0.05).

Because δ power during NREMS is an established marker for sleep homeostasis regulation, we further investigated the genotype effect on this parameter by computing the time course of EEG δ power during NREMS (**Fig. 6**). In accordance with the data presented in Fig. 6, δ power was significantly higher in *Per3*^5/5^ (compared to *Per3*^4/4^) after SD, but only at the beginning of the Rec-L period and during the Rec-D2 period (PROC MIXED *P*<0.05; for *post hoc* contrasts; see Fig. 6, top panel), suggesting a more delayed Rec period in the *Per3*^5/5^ mice. This increase in δ power was not accompanied by changes in the bihourly time course of VSs (Fig. 6, bottom panels). Fragmentation of the VSs was also analyzed, and no changes were found between the *Per3*^5/5^ and the *Per3*^4/4^ mice (data not shown). Thus, the *Per3*^5/5^ mice exhibited 1 of the 3 hallmarks of high NREMS drive: an increase in SWE/δ power, but no increase in NREMS time or consolidation.

### Gene expression in the cortex and hypothalamus after SD

**Figure 7.**
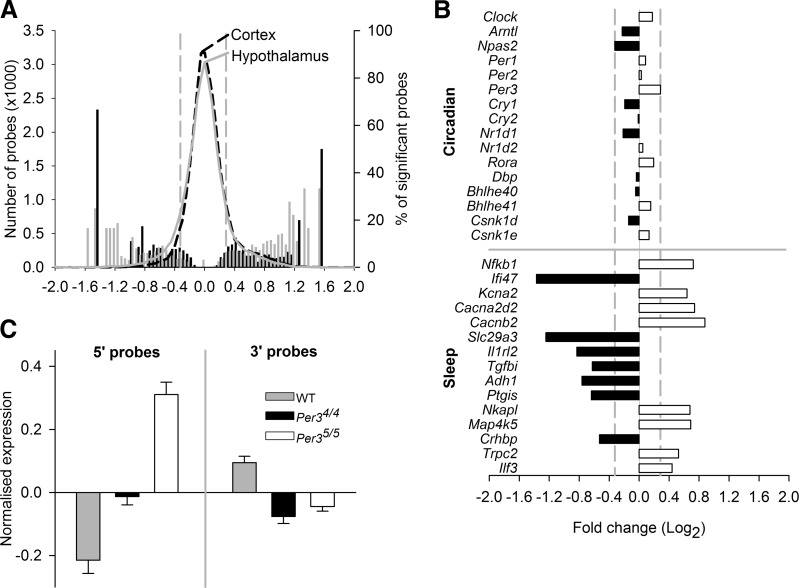
Hypothalamic and cortical gene expression after SD in *Per3*^4/4^ and *Per3*^5/5^ mice. *A*) Distributions (number of probes) of normalized fold change (log_2_) in expression of all detected hypothalamic (gray curve) and cortical (black dashed curve) probes between *Per3*^4/4^ and *Per3*^5/5^. Percentages of detected probes that showed significant differential expression are also indicated on the right *y* axis (gray bars, hypothalamus; black bars, cortex). *B*) Horizontal bars indicate up- and down-regulated fold changes (black, down-regulated in *Per3*^5/5^ *vs. Per3*^4/4^; white, up-regulated in *Per3*^5/5^ *vs. Per3*^4/4^) in the differential expression of a core set of circadian clock genes (top) and a selection of genes associated with sleep regulation (bottom) in both tissues. Vertical dashed gray lines indicate maximum fold change observed in the clock gene transcripts, all nonsignificant (*A*, *B*). *C*) Mean normalized expression in WT (gray), *Per3*^4/4^ (black), and *Per3*^5/5^ (white) of 2 *Homer1* probes that target the 5′ UTR (left) and 2 probes that target the 3′ UTR (right), averaged across both tissues. Error bars = sem.

In the cortex, 816 transcripts showed significant differential expression between the *Per3*^5/5^ and *Per3*^4/4^ mice: 4268 between the *Per3*^5/5^ and WT and 5131 between the *Per3*^4/4^ and WT [**Table 2** and Ancillary Data (gene expression data at http://sleep-sysbio.fhms.surrey.ac.uk/Hasan_14/)]. A similar number of differentially expressed transcripts was observed in the hypothalamus (Table 2 and Ancillary Data). When gene expression between genotypes in both tissues was compared, there were consistently more up-regulated transcripts than down-regulated ones (Table 2 and **Fig. 7*A***).

**Table 2.** Differentially expressed transcripts between genotypes after 12 h SD

Genotypes	Cortex			Hypothalamus		
	DETs	UR	DR	DETs	UR	DR
*Per3*^5/5^ *vs. Per3*^5/5^	816	492	324	716	398	318
*Per3*^5/5^ *vs*. WT	4268	2806	1462	4823	2676	2147
*Per3*^5/5^ *vs*. WT	5131	3213	1918	4138	2286	1852
KI *vs*. WT	3560	2297	1263	3579	1970	1609

DET, differentially expressed transcript; UR, up-regulated; DR, down-regulated.

There were no differences between the *Per3*^5/5^ and the *Per3*^4/4^ mice in the levels of expression in any of the circadian clock genes in either tissue. However, when KI and WT mice were compared, there were differences in the expression of clock genes (Supplemental Table S1). *Clock* was down-regulated in *Per3*^4/4^ compared to WT in both tissues. *Nr1d1* was up-regulated in both KI genotypes compared to WT in the hypothalamus. *Per1* and *Rora* were up-regulated in *Per3*^5/5^ compared with WT mice in the cortex. *Per3*, *Cry2*, *Csnk1d*, and *Bhlhe41* (*Dec2*) were all up-regulated, and *Nr1d2* and *Cry1* were both down-regulated in both KI genotypes compared with their WT expression in both tissues (Fig. 7*B*). Thus, whereas there were no differences in clock gene expression between the KI mice, the presence of the VNTR did affect the expression of some clock genes compared with that in the WT mice.

To investigate sets of significantly differentially expressed genes that could be related to sleep regulation, we ranked differentially expressed transcripts according to their absolute fold change difference between all 3 genotypes in the 2 tissues and analyzed the top 200 transcripts (Ancillary Data). Some sleep-related transcripts were differentially expressed in the *Per3*^5/5^ compared to *Per3*^4/4^ tissues. These included *Nfkb1* (up-regulated in both tissues); *Ifi47* (down-regulated in both tissues); *Kcna2*, *Cacna2d2*, and *Cacnb2* (up-regulated in the hypothalamus); *Slc29a3*, *Il1rl2*, *Tgfbi*, *Adh1*, and *Ptgis* (down-regulated in the hypothalamus); and *Nkapl*, *Map4k5*, *Crhbp*, *Trpc2*, and *Ilf3* (up-regulated in the cortex). Of interest, *Adora1* was up-regulated in the *Per3*^4/4^ mice compared to the WT in both tissues. In the *Per3*^5/5^ mice, *Adora1* did not appear in the top 200 fold-change lists, but it was also significantly up-regulated compared to WT in both tissues (Fig. 7*B*).

In addition to the sleep-related genes that appeared in the top-ranked fold-change lists, we also found significant differential expression for several transcripts of the brain-specific marker for sleep homeostasis *Homer1*. Two probes on the microarray targeted the 5 prime untranslated region (5′ UTR) of 8 mouse *Homer1* transcripts (4 full-length transcripts and 4 shorter transcripts), and they were up-regulated in the KI mice compared to the WT in both tissues and significantly more so in the *Per3*^5/5^ compared to the *Per3*^4/4^ hypothalamus. Two other probes targeted the 3′ UTR of *Homer1* (3 full-length transcripts and 1 short one), and they were down-regulated in both the KI groups, compared with the WT mice in both tissues (Fig. 7*C*).

GO analysis of the transcripts differentially expressed between the *Per3*^4/4^ and *Per3*^5/5^ mice produced almost identical results for both tissues (**Tables 3** and **4**). The top 10 biological processes and molecular functions associated with the differential transcripts included the metabolic process, the macromolecule metabolic process, nucleotide binding, and heterocyclic compound binding. GO analysis of the 1647 transcripts that were differentially regulated in both tissues in both the *Per3*^4/4^ and *Per3*^5/5^ compared to the WT mice in both tissues revealed similar differential expression associated with similar processes and functions (**Table 5**). However, the same analysis also revealed subtle differences between the tissues: the nitrogen compound metabolic process, the cellular nitrogen metabolic process, metal ion binding, and cation binding were enriched only in the hypothalamus, whereas cellular component organization or biogenesis, cellular protein metabolic process, nucleoside phosphate binding, and purine ribonucleoside triphosphate binding were enriched only in the cortex. When the gene expression lists for both tissues were combined, differentially expressed genes between the *Per3*^4/4^ and *Per3*^5/5^ mice were now associated with additional processes and functions that included metabolism and modification focused more on proteins (Table 5).

**Table 3.** GO terms associated with genes differentially regulated between *Per3*^4/4^ and *Per3*^5/5^ in the hypothalamus

GO term	Genes observed	Genes in category	Enrichment ratio	Adjusted *P*
Biological process				
Metabolic	278	9,069	1.42	1.20E-10
Primary metabolic	241	7,662	1.46	1.30E-09
Cellular metabolic	238	7,571	1.46	1.52E-09
Organic substance metabolic	249	8,093	1.43	2.39E-09
Single-organism metabolic	253	8,434	1.39	1.99E-08
Cellular	356	13,317	1.24	6.37E-08
Macromolecule metabolic	195	6,456	1.40	1.05E-05
Cellular biosynthetic	137	4,141	1.54	1.71E-05
Organic substance biosynthetic	139	4,245	1.52	2.19E-05
Cellular macromolecule metabolic	174	5,669	1.42	2.23E-05
Molecular function				
Binding	327	10,881	1.41	1.91E-15
Protein binding	194	6,206	1.46	1.65E-07
Catalytic activity	164	5,115	1.50	6.64E-07
Ion binding	167	5,230	1.50	6.64E-07
Organic cyclic compound binding	145	4,641	1.46	2.68E-05
Heterocyclic compound binding	144	4,592	1.47	2.68E-05
Nucleotide binding	82	2,268	1.69	6.74E-05
Nucleoside phosphate binding	82	2,268	1.69	6.74E-05
Metal ion binding	112	3,443	1.52	8.71E-05
Small-molecule binding	86	2,445	1.65	8.71E-05

**Table 4.** GO terms associated with genes differentially regulated between *Per3*^4/4^ and *Per3*^5/5^ in the cortex

GO term	Genes observed	Genes in category	Enrichment ratio	Adjusted *P*
Biological process				
Metabolic	333	9,069	1.53	9.72E-20
Cellular metabolic	291	7,571	1.60	7.60E-19
Organic substance metabolic	304	8,093	1.56	9.12E-19
Single-organism metabolic	310	8,434	1.53	6.60E-18
Primary metabolic	289	7,662	1.57	1.08E-17
Cellular macromolecule metabolic	231	5,669	1.70	1.40E-16
Macromolecule metabolic	248	6,456	1.60	5.54E-15
Organic cyclic compound metabolic	194	4,672	1.73	6.72E-14
Heterocycle metabolic	186	4,500	1.72	5.68E-13
Cellular aromatic compound metabolism	186	4,521	1.71	8.15E-13
Molecular function				
Binding	379	10,881	1.44	8.20E-21
Organic cyclic compound binding	200	4,641	1.78	2.71E-16
Heterocyclic compound binding	199	4,592	1.79	2.71E-16
Small-molecule binding	122	2,445	2.06	3.95E-13
Nucleotide binding	114	2,268	2.08	1.64E-12
Nucleoside phosphate binding	114	2,268	2.08	1.64E-12
Ion binding	201	5,230	1.59	2.04E-11
Anion binding	105	2,264	1.92	2.21E-09
RNA binding	50	781	2.65	2.01E-08
Purine nucleoside binding	84	1,740	2.00	3.41E-08

**Table 5.** GO terms associated with genes differentially regulated between *Per3*^4/4^ and *Per3*^5/5^ in both the hypothalamus and cortex

GO term	Probes observed	Enrichment score	Percentage of category	Adjusted *P*
Biological process				
Cellular protein modification process	21	2.89	25.93	1.10E-03
Protein modification process	21	2.89	25.93	1.10E-03
Macromolecule modification	22	2.92	27.16	1.10E-03
Cellular protein metabolic process	23	2.4	28.40	4.20E-03
Protein metabolic process	26	2.24	32.10	4.20E-03
Phosphatidylinositol phosphorylation	3	29.6	3.70	7.40E-03
Metabolic process	46	1.55	56.79	7.40E-03
Phosphorylation	14	2.95	17.28	1.16E-02
Lipid phosphorylation	3	25.49	3.70	1.16E-02
Primary metabolic process	40	1.6	49.38	1.56E-02
Molecular function				
Small molecule binding	18	2.26	22.22	1.79E-02
Nucleotide binding	17	2.31	20.99	1.79E-02
Anion binding	17	2.31	20.99	1.79E-02
Nucleoside phosphate binding	17	2.31	20.99	1.79E-02
Binding	50	1.41	61.73	1.79E-02
Glycoprotein binding	3	13.18	3.70	1.99E-02
Purine nucleotide binding	14	2.43	17.28	1.99E-02
Ribonucleotide binding	14	2.42	17.28	1.99E-02
Purine nucleotide binding	14	2.44	17.28	1.99E-02
Purine nucleoside binding	13	2.3	16.05	2.44E-02

### Gene expression in the hypothalamus during behavioral suppression by light

Behavioral suppression by light in the ultradian protocol was not significantly different between any of the genotypes (data not shown). Fold changes in hypothalamic gene expression between the KI and WT mice exhibited normal distributions (Supplemental Fig. S1*A*). From these distributions, 282 probes were differentially expressed between the *Per3*^5/5^ and WT mice, and 96 probes were differentially expressed between the *Per3*^4/4^ and WT mice (Supplemental Fig. S1*A*, see Supplemental Table S2 for top 30-fold change probes for each genotype). This analysis showed that none of the core set of circadian clock gene transcripts was differentially expressed between the WT and KI tissues (Supplemental Fig. S1*B*). By contrast, among the probes that were differentially expressed were genes of interest for sleep homeostasis, including *Homer1*, *Ptgs2*, and *Tnfrsf25* (all down-regulated in the *Per3*^5/5^ compared to the WT tissues); *Kcne2* (down-regulated in *Per3*^5/5^ and up-regulated in *Per3*^4/4^); and *Prl* (down-regulated in *Per3*^4/4^ and up-regulated in *Per3*^5/5^). A further 7 genes (*Ccl21a*, *Slx1b*, *Rpp40*, *Ttr*, *Tmem72*, *Sln*, and *Apoc4*) were differentially expressed in both the *Per3*^4/4^ and *Per3*^5/5^ tissues. *Ccl21a* (a chemokine ligand) was down-regulated in tissues of both the KI groups and was the most down-regulated probe in the *Per3*^4/4^ tissue. Gene enrichment and functional annotation analysis revealed that the 282 differentially expressed probes in the *Per3*^5/5^ hypothalamic tissue were significantly associated with biological processes and molecular functions that included terms linked with neuronal development and signaling, as well as the regulation of transcription (Supplemental Fig. S1*C*). There were no significant GO terms associated with the differentially expressed probes in the *Per3*^4/4^ mice, because of the smaller number of probes analyzed.

## DISCUSSION

Our data show that expression of the primate-specific 4- or 5-repeat *PER3* VNTR polymorphism in mice altered the time course of two established EEG markers of sleep homeostasis: θ activity during wakefulness and δ power during NREMS. By contrast, no genotype differences were observed between the KI mice in measures of circadian period and responses to light or in the expression levels of transcripts of the core set of circadian clock genes. Moreover, insertion of the VNTR associates with differential expression of genes linked with sleep regulatory processes, including an established marker of sleep homeostasis, *Homer1*.

The current mouse data showing that the number of tandem repeats does not change circadian periodicity is in line with previous mouse data showing no major effect of *Per3* KO (11, 13) and with human data showing that the *PER3* VNTR affects neither the *in vitro* nor *in vivo* period when assessed in dim light (27). Also, extensive whole-genome microarray analyses showed no difference between hypothalamic and cortical expression of circadian clock genes between the two KI genotypes after SD and also during an ultradian LD protocol, whereas we observed significant differences in the expression of genes associated with sleep. These included *Homer1* and *Ptgs2* (39), *Prl* (40), *Kcna2* (41), and *Adh1* (42, 43), whereas *Adora1* (44) was up-regulated in both the hypothalamic and cortical tissues in the 2 KI groups compared with the WT.

In response to SD, GO analysis of differential gene expression identified processes and functions that are typically affected by sleep loss, such as macromolecular metabolic processes. During the ultradian protocol, where there was no behavioral phenotype, genes differentially expressed between the *Per3*^5/5^ and WT mice in the hypothalamus were associated with GO terms that included neuronal development and signaling. The latter finding is of interest because of the individual differences in cognitive performance and brain function that are associated with the human *PER3* VNTR (22–24). Therefore, taken together, these humanized mouse data are consistent with existing human data that suggest that the *PER3* VNTR does not affect the circadian clock in the hypothalamus, but is associated with differences in homeostatic sleep regulation and cognitive vulnerability to sleep loss and in mice with the differential expression of transcripts that regulate and respond to sleep and neuronal development and function.

*Homer1a* has been described as a brain-specific marker of sleep homeostasis because its expression increases in response to SD (39). In the current study, *Homer1* expression was increased in the two KI genotypes compared with the WT, more so in the *Per3*^5/5^ mice in both tissues in the SD and ultradian protocols. However, this was true only of probes that targeted the 5′ UTR of *Homer1* transcripts; probes that targeted the 3′ UTR showed down-regulation compared to the WT in both the KI genotypes. This result suggests that there is up-regulated expression of nascent mRNA for *Homer1* in response to SD but reduced levels of alternatively spliced transcripts.

As far as we are aware, this is the first time that a common human polymorphism involved in sleep homeostasis has been inserted into an animal model. A rare familial single-nucleotide mutation in *DEC2* regulates sleep duration in humans, and insertion of this mutation in mice also affects sleep duration in an SD-dependent way (45). The mutation that is linked with familial advanced sleep phase disorder (FASPD) has been reproduced in transgenic mice, but only with regard to circadian phenotype; the effects on sleep EEG were not assessed (46). An intronic SNP in the potassium channel gene *ABCC9* was found to be associated with short periods of sleep in humans, and silencing of the homologue gene in Drosophila created a sleepless phenotype in the first part of the night (47). However, although that study clearly suggests a role for *ABCC9* in sleep regulation, it did not identify the causative genetic marker.

The lack of an effect of genotype on BSL VSs in the current study contrasts with some previous studies in clock gene transgenic mice. For example, *Clock*-mutant mice showed a reduced BSL sleep duration (48), *Npas2*-KO mice had reduced spindle frequency activity in NREMS (49), and *Cry1/Cry2* double-KO mice had increased NREMS bout length and NREM δ power (8). It should be noted that, in the current study, *Cry1* expression was highly down-regulated in the KI mice compared with WT mice in both the hypothalamus and cortex after SD. Overall, this observation implies that the VNTR-mediated effects on sleep are observed only when sleep is manipulated.

The higher EEG θ activity in wakefulness observed in the *Per3*^5/5^ mice during forced wakefulness and the genotype difference in BSL θ power during REMS observed in the *Per3*^5/5^ mice are in line with the higher θ/alpha power seen in *PER3*^5/5^ humans, at BSL and during SD (21).

The observation that during sleep Rec, the NREM δ power was higher in the *Per3*^5/5^ mice is also consistent with human data from *PER3*^5/5^ individuals (21). In addition, the *Per3*^5/5^ mice had a greater and more complete (*i.e*., return to BSL level) sleep homeostatic response, as quantified by the accumulation of NREM δ power, when compared to the *Per3*^4/4^ mice. In addition, in a previous human protocol of sleep restriction/deprivation, NREM δ power was found to be higher in the *PER3*^5/5^ genotype compared to the *PER3*^4/4^ (22). These consistent observations between species are remarkable, given that the only difference between these genotypes is the presence of either the 4- or 5-repeat VNTR alleles.

## Supplementary Material

Supplemental Data

## References

[1] Rusterholz T. Durr R. Achermann P. (2010) Inter-individual differences in the dynamics of sleep homeostasis. Sleep 33, 491–498 20394318 10.1093/sleep/33.4.491 PMC2849788

[2] Adan A. Archer S. N. Hidalgo M. P. Di Milia L. Natale V. Randler C. (2012) Circadian typology: a comprehensive review. Chronobiol. Int. 29, 1153–1175 23004349 10.3109/07420528.2012.719971

[3] Borbely A. A. (1982) A two process model of sleep regulation. Hum. Neurobiol. 1, 195–204 7185792

[4] Dijk D. J. Czeisler C. A. (1995) Contribution of the circadian pacemaker and the sleep homeostat to sleep propensity, sleep structure, electroencephalographic slow waves, and sleep spindle activity in humans. J. Neurosci. 15, 3526–3538 7751928 10.1523/JNEUROSCI.15-05-03526.1995 PMC6578184

[5] Buhr E. D. Takahashi J. S. (2013) Molecular components of the mammalian circadian clock. Handb. Exp. Pharmacol. 217, 3–27 23604473 10.1007/978-3-642-25950-0_1 PMC3762864

[6] Franken P. Dijk D. J. (2009) Circadian clock genes and sleep homeostasis. Eur. J. Neurosci. 29, 1820–1829 19473235 10.1111/j.1460-9568.2009.06723.x

[7] Franken P. Chollet D. Tafti M. (2001) The homeostatic regulation of sleep need is under genetic control. J. Neurosci. 21, 2610–2621 11306614 10.1523/JNEUROSCI.21-08-02610.2001 PMC6762509

[8] Wisor J. P. O'Hara B. F. Terao A. Selby C. P. Kilduff T. S. Sancar A. Edgar D. M. Franken P. (2002) A role for cryptochromes in sleep regulation. BMC Neurosci. 3, 20 12495442 10.1186/1471-2202-3-20 PMC149230

[9] Allebrandt K. V. Teder-Laving M. Akyol M. Pichler I. Muller-Myhsok B. Pramstaller P. Merrow M. Meitinger T. Metspalu A. Roenneberg T. (2010) CLOCK gene variants associate with sleep duration in two independent populations. Biol. Psychiatry 67, 1040–1047 20149345 10.1016/j.biopsych.2009.12.026

[10] Yagita K. Yamaguchi S. Tamanini F. van Der Horst G. T. Hoeijmakers J. H. Yasui A. Loros J. J. Dunlap J. C. Okamura H. (2000) Dimerization and nuclear entry of mPER proteins in mammalian cells. Genes Dev. 14, 1353–1363 10837028 PMC316664

[11] Shearman L. P. Jin X. Lee C. Reppert S. M. Weaver D. R. (2000) Targeted disruption of the mPer3 gene: subtle effects on circadian clock function. Mol. Cell. Biol. 20, 6269–6275 10938103 10.1128/mcb.20.17.6269-6275.2000 PMC86101

[12] Bae K. Jin X. Maywood E. S. Hastings M. H. Reppert S. M. Weaver D. R. (2001) Differential functions of mPER1, mPER2, and mPER3 in the SCN circadian clock. Neuron 30, 525–536 11395012 10.1016/s0896-6273(01)00302-6

[13] Van der Veen D. R. Archer S. N. (2010) Light-dependent behavioral phenotypes in PER3-deficient mice. J. Biol. Rhythms 25, 3–8 20075295 10.1177/0748730409356680

[14] Pendergast J. S. Niswender K. D. Yamazaki S. (2012) Tissue-specific function of Period3 in circadian rhythmicity. PLoS One 7, e30254 22253927 10.1371/journal.pone.0030254 PMC3256228

[15] Hasan S. van der Veen D. R. Winsky-Sommerer R. Dijk D. J. Archer S. N. (2011) Altered sleep and behavioral activity phenotypes in PER3-deficient mice. Am. J. Physiol. Regul. Integr. Comp. Physiol. 301, R1821–R1830 21957163 10.1152/ajpregu.00260.2011

[16] Archer S. N. Robilliard D. L. Skene D. J. Smits M. Williams A. Arendt J. von Schantz M. (2003) A length polymorphism in the circadian clock gene Per3 is linked to delayed sleep phase syndrome and extreme diurnal preference. Sleep 26, 413–415 12841365 10.1093/sleep/26.4.413

[17] Pereira D. S. Tufik S. Louzada F. M. Benedito-Silva A. A. Lopez A. R. Lemos N. A. Korczak A. L. D'Almeida V. Pedrazzoli M. (2005) Association of the length polymorphism in the human Per3 gene with the delayed sleep-phase syndrome: does latitude have an influence upon it? Sleep 28, 29–32 15700718

[18] Jones K. H. Ellis J. von Schantz M. Skene D. J. Dijk D. J. Archer S. N. (2007) Age-related change in the association between a polymorphism in the PER3 gene and preferred timing of sleep and waking activities. J. Sleep Res. 16, 12–16 17309758 10.1111/j.1365-2869.2007.00561.x PMC7611878

[19] Lazar A. S. Slak A. Lo J. C. Santhi N. von Schantz M. Archer S. N. Groeger J. A. Dijk D. J. (2012) Sleep, diurnal preference, health, and psychological well-being: a prospective single-allelic-variation study. Chronobiol. Int. 29, 131–146 22324552 10.3109/07420528.2011.641193

[20] Viola A. U. Archer S. N. James L. M. Groeger J. A. Lo J. C. Skene D. J. von Schantz M. Dijk D. J. (2007) PER3 polymorphism predicts sleep structure and waking performance. Curr. Biol. 17, 613–618 17346965 10.1016/j.cub.2007.01.073

[21] Goel N. Banks S. Mignot E. Dinges D. F. (2009) PER3 polymorphism predicts cumulative sleep homeostatic but not neurobehavioral changes to chronic partial sleep deprivation. PLoS One 4, e5874 19516903 10.1371/journal.pone.0005874 PMC2689932

[22] Groeger J. A. Viola A. U. Lo J. C. von Schantz M. Archer S. N. Dijk D. J. (2008) Early morning executive functioning during sleep deprivation is compromised by a PERIOD3 polymorphism. Sleep 31, 1159–1167 18714788 PMC2542962

[23] Vandewalle G. Archer S. N. Wuillaume C. Balteau E. Degueldre C. Luxen A. Maquet P. Dijk D. J. (2009) Functional magnetic resonance imaging-assessed brain responses during an executive task depend on interaction of sleep homeostasis, circadian phase, and PER3 genotype. J. Neurosci. 29, 7948–7956 19553435 10.1523/JNEUROSCI.0229-09.2009 PMC6666044

[24] Lo J. C. Groeger J. A. Santhi N. Arbon E. L. Lazar A. S. Hasan S. von Schantz M. Archer S. N. Dijk D. J. (2012) Effects of partial and acute total sleep deprivation on performance across cognitive domains, individuals and circadian phase. PLoS One 7, e45987 23029352 10.1371/journal.pone.0045987 PMC3454374

[25] Archer S. N. Viola A. U. Kyriakopoulou V. von Schantz M. Dijk D. J. (2008) Inter-individual differences in habitual sleep timing and entrained phase of endogenous circadian rhythms of BMAL1, PER2 and PER3 mRNA in human leukocytes. Sleep 31, 608–617 18517031 10.1093/sleep/31.5.608 PMC2398752

[26] Viola A. U. Chellappa S. L. Archer S. N. Pugin F. Götz T. Dijk D. J. Cajochen C. (2012) Interindividual differences in circadian rhythmicity and sleep homeostasis in older people: effect of a PER3 polymorphism. Neurobiol. Aging 33, 1010.e17–1010.e27 22169200 10.1016/j.neurobiolaging.2011.10.024

[27] Hasan S. Santhi N. Lazar A. S. Slak A. Lo J. von Schantz M. Archer S. N. Johnston J. D. Dijk D. J. (2012) Assessment of circadian rhythms in humans: comparison of real-time fibroblast reporter imaging with plasma melatonin. FASEB J. 26, 2414–2423 22371527 10.1096/fj.11-201699 PMC3360144

[28] Dijk D. J. Archer S. N. (2010) PERIOD3, circadian phenotypes, and sleep homeostasis. Sleep Med. Rev. 14, 151–160 19716732 10.1016/j.smrv.2009.07.002

[29] Mongrain V. La Spada F. Curie T. Franken P. (2011) Sleep loss reduces the DNA-binding of BMAL1, CLOCK, and NPAS2 to specific clock genes in the mouse cerebral cortex. PLoS One 6, e26622 22039518 10.1371/journal.pone.0026622 PMC3200344

[30] Saper C. B. Cano G. Scammell T. E. (2005) Homeostatic, circadian, and emotional regulation of sleep. J. Comp. Neurol. 493, 92–98 16254994 10.1002/cne.20770

[31] Ebisawa T. Uchiyama M. Kajimura N. Mishima K. Kamei Y. Watanabe T. Sekimoto M. Shibui K. Kim K. Kudo Y. Ozeki Y. Sugishita M. Toyoshima R. Inoue Y. Yamada N. Nagase T. Ozaki N. Ohara O. Ishida N. Okawa M. Takahashi K. Yamauchi T. (2001) Association of structural polymorphisms in the human period3 gene with delayed sleep phase syndrome. EMBO Rep. 2, 342–346 11306557 10.1093/embo-reports/kve070 PMC1083867

[32] Köntgen F. Süss G. Stewart C. Steinmetz M. Bluethmann H. (1993) Targeted disruption of the MHC class II Aa gene in C57BL/6 mice. Int. Immunol. 5, 957–964 8398989 10.1093/intimm/5.8.957

[33] Hughes E. D. Qu Y. Y. Genik S. J. Lyons R. H. Pacheco C. D. Lieberman A. P. Samuelson L. C. Nasonkin I. Q. Camper S. A. Van Keuren M. L. Saunders T. L. (2007) Genetic variation in C57BL/6 ES cell lines and genetic instability in the Bruce4 C57BL/6 ES cell line. Mamm. Genome 18, 549–558 17828574 10.1007/s00335-007-9054-0

[34] Sokolove P. G. Bushell W. N. (1978) The chi square periodogram: its utility for analysis of circadian rhythms. J. Theor. Biol. 72, 131–160 566361 10.1016/0022-5193(78)90022-x

[35] Franken P. Malafosse A. Tafti M. (1998) Genetic variation in EEG activity during sleep in inbred mice. Am. J. Physiol. Regul. Integr. Comp. Physiol. 275, R1127–R1137 10.1152/ajpregu.1998.275.4.R1127 9756543

[36] Laing E. Smith C. P. (2010) RankProdIt: a web-interactive rank products analysis tool. BMC Res. Notes 3, 221 20691047 10.1186/1756-0500-3-221 PMC2930644

[37] Duncan D. Prodduturi N. Zhang B. (2010) WebGestalt2: an updated and expanded version of the web-based gene set analysis toolkit. BMC Bioinformatics 11, P10

[38] Aschoff J. (1960) Exogenous and endogenous components in circadian rhythms. Cold Spring Harb. Symp. Quant. Biol. 25, 11–28 13684695 10.1101/sqb.1960.025.01.004

[39] Maret S. Dorsaz S. Gurcel L. Pradervand S. Petit B. Pfister C. Hagenbuchle O. O'Hara B. F. Franken P. Tafti M. (2007) Homer1a is a core brain molecular correlate of sleep loss. Proc. Natl. Acad. Sci. U.S.A. 104, 20090–20095 18077435 10.1073/pnas.0710131104 PMC2148427

[40] Obál F Jr. Garcia-Garcia F. Kacsóh B. Taishi P. Bohnet S. Horseman N. D. Krueger J. M. (2005) Rapid eye movement sleep is reduced in prolactin-deficient mice. J. Neurosci. 25, 10282–10289 16267236 10.1523/JNEUROSCI.2572-05.2005 PMC6725790

[41] Douglas C. L. Vyazovskiy V. Southard T. Chiu S. Y. Messing A. Tononi G. Cirelli C. (2007) Sleep in Kcna2 knockout mice. BMC Biol. 5, 42 17925011 10.1186/1741-7007-5-42 PMC2151933

[42] Deltour L. Foglio M. H. Duester G. (1999) Metabolic deficiencies in alcohol dehydrogenase Adh1, Adh3, and Adh4 null mutant mice. Overlapping roles of Adh1 and Adh4 in ethanol clearance and metabolism of retinol to retinoic acid. J. Biol. Chem. 274, 16796–16801 10358022 10.1074/jbc.274.24.16796

[43] Maret S. Franken P. Dauvilliers Y. Ghyselinck N. B. Chambon P. Tafti M. (2005) Retinoic acid signaling affects cortical synchrony during sleep. Science 310, 111–113 16210540 10.1126/science.1117623

[44] Thakkar M. M. Engemann S. C. Walsh K. M. Sahota P. K. (2008) Adenosine and the homeostatic control of sleep: effects of A1 receptor blockade in the perifornical lateral hypothalamus on sleep-wakefulness. Neuroscience 153, 875–880 18440150 10.1016/j.neuroscience.2008.01.017

[45] He Y. Jones C. R. Fujiki N. Xu y. Guo B. Holder J. L Jr. Rossner M. J. Nishino S. Fu Y. H. (2009) The transcriptional repressor DEC2 regulates sleep length in mammals. Science 325, 866–870 19679812 10.1126/science.1174443 PMC2884988

[46] Xu Y. Toh K. L. Jones C. R. Shin J. Y. Fu Y. H. Ptacek L. J. (2007) Modeling of a human circadian mutation yields insights into clock regulation by PER2. Cell 128, 59–70 17218255 10.1016/j.cell.2006.11.043 PMC1828903

[47] Allebrandt K. V. Amin N. Müller-Myhsok B. Esko T. Teder-Laving M. Azevedo R. V. Hayward C. van Mill J. Vogelzangs N. Green E. W. Melville S. A. Lichtner P. Wichmann H. E. Oostra B. A. Janssens A. C. Campbell H. Wilson J. F. Hicks A. A. Pramstaller P. P. Dogas Z. Rudan I. Merrow M. Penninx B. Kyriacou C. P. Metspalu A. van Duijn C. M. Meitinger T. Roenneberg T. (2013) A K(ATP) channel gene effect on sleep duration: from genome-wide association studies to function in Drosophila. Mol. Psychiatry 18, 122–132 22105623 10.1038/mp.2011.142

[48] Naylor E. Bergmann B. M. Krauski K. Zee P. C. Takahashi J. S. Vitaterna M. H. Turek F. W. (2000) The circadian clock mutation alters sleep homeostasis in the mouse. J. Neurosci. 20, 8138–8143 11050136 10.1523/JNEUROSCI.20-21-08138.2000 PMC6772726

[49] Franken P. Dudley C. A. Estill S. J. Barakat M. Thomason R. O'Hara B. F. McKnight S. L. (2006) NPAS2 as a transcriptional regulator of non-rapid eye movement sleep: genotype and sex interactions. Proc. Natl. Acad. Sci. U.S.A. 103, 7118–7123 16636276 10.1073/pnas.0602006103 PMC1459027

