# Supplementary material for: A human sleep homeostasis phenotype in mice expressing a primate-specific PER3 variable-number tandem-repeat coding-region polymorphism
Source: FASEB J. 2014 Jun;28(6):2441–54. doi: 10.1096/fj.13-240135 (PMC4046067; doi:10.1096/fj.13-240135)
Supplement: Supplemental Data [file supp_28_6_2441__index.html]

A human sleep homeostasis phenotype in mice expressing a primate-specific PER3 variable-number tandem-repeat coding-region polymorphism — A human sleep homeostasis phenotype in mice expressing a primate-specific PER3 variable-number tandem-repeat coding-region polymorphism — A human sleep homeostasis phenotype in mice expressing a primate-specific PER3 variable-number tandem-repeat coding-region polymorphism — Supplemental Data 

# A human sleep homeostasis phenotype in mice expressing a primate-specific *PER3* variable-number tandem-repeat coding-region polymorphism

## Supplemental Data

**Files in this Data Supplement:**

- Supplemental Data - (*13-240135SuppData.pdf; 415 KB*)
