# Supplementary material for: A human sleep homeostasis phenotype in mice expressing a primate-specific PER3 variable-number tandem-repeat coding-region polymorphism
Source: FASEB J. 2014 Jun;28(6):2441–54. doi: 10.1096/fj.13-240135 (PMC4046067; doi:10.1096/fj.13-240135)
Supplement: Supplemental Data [file supp_fj.13-240135_13-240135SuppData.pdf]

A

B

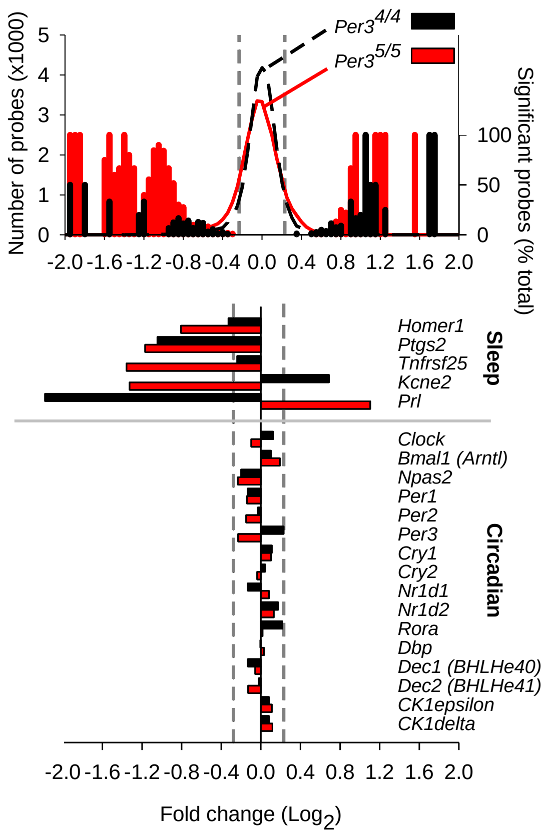

C

		Observed probes	Enrichment score	Percentage of category	Adjusted P value
Biological process	behavior	16	4.46	8.65	3.00E-04
	multicellular organismal signaling	16	4.25	8.65	3.00E-04
	cellular component morphogenesis	19	3.65	10.27	3.00E-04
	generation of neurons	20	3.4	10.81	3.00E-04
	neuron projection development	15	4.38	8.11	3.00E-04
	cell morphogenesis	18	3.74	9.73	3.00E-04
	neurogenesis	21	3.33	11.35	3.00E-04
	synaptic transmission	14	4.87	7.57	3.00E-04
	transmission of nerve impulse	15	4.15	8.11	4.00E-04
	neuron development	16	3.89	8.65	4.00E-04
Molecular function	receptor binding	21	2.77	11.35	4.70E-03
	RNA pol. II sequence specific binding	5	9.32	2.70	7.00E-03
	retinol dehydrogenase activity	3	25.8	1.62	7.00E-03
	protein binding	62	1.55	33.51	7.00E-03
	transcription regulatory region DNA binding	9	4.2	4.86	7.00E-03
	RNA pol. II regulatory DNA binding	5	8.5	2.70	7.00E-03
	protein dimerization activity	16	2.65	8.65	7.00E-03
	regulatory region nucleic acid binding	9	4.1	4.86	7.00E-03
	sequence specific regulatory binding	6	6.45	3.24	7.00E-03
	GTPase activator activity	7	5.44	3.78	7.00E-03
Cellular component	neuron projection	19	3.71	10.27	1.00E-04
	extracellular region	30	2.53	16.22	1.00E-04
	cell projection	25	2.77	13.51	2.00E-04
	postsynaptic membrane	8	6.72	4.32	8.00E-04
	postsynaptic density	6	7.99	3.24	8.00E-04
	neuronal cell body	11	4.18	5.95	8.00E-04
	intracellular	98	1.33	52.97	8.00E-04
	cell	10	1.28	5.41	8.00E-04
	axon	10	4.72	5.41	8.00E-04
	synaptic membrane	8	5.69	4.32	8.00E-04

Figure S1. Hypothalamic gene expression in transgenic compared to WT mice. (A) The distributions (number of probes) of normalized fold change (Log_2) in expression of all detected hypothalamic probes between *Per3*$^{4/4}$ compared to WT (black line) and *Per3*$^{5/5}$ compared to WT (red line). The percentage of these detected probes that were significantly differentially expressed are also indicated with the right-hand axis (black bars = *Per3*$^{4/4}$, red bars = *Per3*$^{5/5}$). (B) Horizontal bars (black = *Per3*$^{4/4}$, red = *Per3*$^{5/5}$) indicate the up- and down-regulated fold changes (Log_2) in the differential expression (compared to WT) of five genes associated with sleep homeostasis (top), and a core set of circadian clock genes (bottom). The vertical grey dashed lines in panels A and B indicate the maximal fold change observed in the clock gene transcripts, which were all non-significant. (C) Gene ontology analysis of biological process, molecular function, and cellular component terms that were associated with differentially expressed hypothalamic genes in *Per3*$^{5/5}$ compared with WT (number of observed probes contributing to each term, enrichment score, percentage of differentially expressed genes contributing to each term and adjusted P value are indicated). Gene ontology analysis did not reveal significant terms for the differential expression in the *Per3*$^{4/4}$ mice due to the smaller number of probes analyzed.

ProbeName	GeneSymbol	$Per3^{4/4}$ vs. $Per3^{5/5}$	WT vs $Per3^{4/4}$ and/or $Per3^{5/5}$	$Per3^{5/5}$	$Per3^{4/4}$	WT
A_52_P267651	*Arntl*			-0.12898	0.10941	-0.04331
A_51_P272553	*Bhlhe40*			-0.01092	-0.04802	0.04374
A_51_P451176	*Bhlhe41*		P = 0.0052	0.16994	0.06284	-0.31015
A_52_P618912	*Bhlhe41*			0.26348	0.07570	-0.23859
A_52_P161365	*Clock*			0.11360	-0.15249	-0.01458
A_52_P279391	*Clock*			0.11598	-0.07711	0.00522
A_52_P334670	*Clock*		P = 0.0262	0.05596	-0.14214	0.29667
A_52_P334677	*Clock*			-0.05199	-0.14977	0.20162
A_51_P302204	*Cry1*			-0.25761	0.06965	-0.06256
A_52_P420500	*Cry1*		P = 0.0458	-0.68362	-0.50719	0.31876
A_51_P135542	*Cry2*		P = 0.0055	0.00655	0.08840	-0.49925
A_52_P248378	*Cry2*		P = 0.0434	0.06761	0.06106	-0.16752
A_51_P136277	*Csnk1d*		P = 0.0245	0.09405	0.32989	-0.33567
A_51_P359813	*Csnk1d*			-0.12066	-0.04088	0.10839
A_52_P11576	*Csnk1d*			0.11080	0.18629	-0.16877
A_52_P477369	*Csnk1d*		P = 0.0161	0.22056	0.36733	-0.37472
A_51_P429197	*Csnk1e*			0.16857	-0.02134	-0.01003
A_52_P542419	*Csnk1e*			-0.00284	0.04857	0.00023
A_51_P180492	*Dbp*		P = 0.0102	0.02481	0.08902	-0.29256
A_52_P534749	*Npas2*		P = 0.6067	-0.00435	-0.06607	-0.17249
A_51_P223776	*Nr1d1*		P = 0.0245	0.08307	0.12696	-0.46491
A_52_P130952	*Nr1d1*			0.11137	0.30631	-0.36408
A_52_P130961	*Nr1d1*			-0.41301	0.11641	-0.34571
A_52_P303891	*Nr1d2*		P = 0.0363	-0.08817	-0.09040	0.23406
A_52_P79889	*Per1*			0.09700	0.02805	-0.01378
A_51_P282760	*Per2*			-0.09000	-0.02738	0.09985
A_52_P536869	*Per2*			-0.12907	0.32956	-0.13785
A_51_P258493	*Per3*		P = 0.0372	0.25613	-0.03558	-0.21079
A_51_P477779	*Rora*			0.15948	-0.09179	0.16905
A_52_P652442	*Rora*			0.23779	-0.04323	0.01108
A_52_P652950	*Rora*			0.35369	-0.05030	0.04104

Supplemental table S1A: Clock gene expression in hypothalamic samples

ProbeName	GeneSymbol	$Per3^{4/4}$ vs.$Per3^{5/5}$	WT vs $Per3^{4/4}$ and/or $Per3^{5/5}$	$Per3^{5/5}$	$Per3^{4/4}$	WT
A_52_P267651	*Arntl*			-0.06289	0.14908	-0.01202
A_51_P272553	*Bhlhe40*			-0.02212	-0.07854	-0.06550
A_51_P451176	*Bhlhe41*		P = 0.0032	0.18719	0.05677	-0.24150
A_52_P618912	*Bhlhe41*			0.09504	0.14551	-0.26483
A_52_P161365	*Clock*			0.08955	-0.02715	0.05125
A_52_P279391	*Clock*			0.07064	-0.15654	-0.07816
A_52_P334670	*Clock*			0.05231	-0.09172	0.21352
A_52_P334677	*Clock*		P = 0.0377	-0.02105	-0.19695	0.10035
A_51_P302204	*Cry1*			-0.05304	0.16666	-0.09487
A_52_P420500	*Cry1*		P = 0.0071	-0.53089	-0.47892	0.57222
A_51_P135542	*Cry2*		P = 0.0017	0.26195	0.13782	-0.45281
A_52_P248378	*Cry2*		P = 0.0078	0.08495	0.18414	-0.16797
A_51_P136277	*Csnk1d*		P = 0.0094	0.01389	0.27917	-0.39669
A_51_P359813	*Csnk1d*			-0.01190	-0.04583	-0.01672
A_52_P11576	*Csnk1d*		P = 0.0019	0.01862	0.20343	-0.43821
A_52_P477369	*Csnk1d*		P = 0.0015	0.02986	0.20707	-0.64890
A_51_P429197	*Csnk1e*			0.15034	-0.05504	0.00226
A_52_P542419	*Csnk1e*			-0.02476	-0.03711	-0.01421
A_51_P180492	*Dbp*			0.09361	0.10192	-0.18879
A_52_P79889	*Npas2*		P = 0.0418	0.13054	0.03951	-0.21103
A_51_P223776	*Nr1d1*			0.17853	0.19377	-0.22922
A_52_P130952	*Nr1d1*			-0.01569	0.24713	-0.35349
A_52_P130961	*Nr1d1*			-0.22156	0.01417	-0.34439
A_52_P303891	*Nr1d2*		P = 0.0109	0.00165	-0.09362	0.13994
A_52_P79889	*Per1*		P = 0.0412	0.14551	0.04336	-0.12888
A_51_P282760	*Per2*			0.10941	-0.02899	-0.01415
A_52_P536869	*Per2*			0.03497	0.22631	-0.15680
A_51_P258493	*Per3*		P = 0.008	0.22901	-0.04939	-0.45997
A_51_P477779	*Rora*			0.00057	-0.16541	0.01888
A_52_P652442	*Rora*			0.05234	-0.04869	-0.05583
A_52_P652950	*Rora*			0.10809	-0.05947	-0.07725

Supplemental table S1B: Clock gene expression in cortical samples

Per3$^{4/4}$		*Per3*$^{5/5}$	
**Gene/Probe**	**Fold change**	**Gene/Probe**	**Fold change**
Ccl21a	-3.39	*Lct*	-3.38
Gdpd3	2.76	*A_52_P788961*	2.81
Lcn12	-2.32	*Grn*	-2.38
Pttg1	2.31	*8430429K09Rik*	2.25
Prl	-2.18	*Dsp*	-2.20
Dyrk4	-2.17	*Neurod6*	-1.98
LOC674214	-1.95	*Sln*	-1.96
Slx1b	-1.81	*A_51_P311235*	-1.90
Ugt2b38	-1.78	*Agxt*	-1.87
Apoc4	1.77	*Hamp*	-1.62
Tsix	1.68	*Neurod2*	-1.62
Gm3893	-1.56	*Thbs4*	-1.61
Wfdc2	1.27	*Ccl21a*	-1.57
A930018M24Rik	-1.23	*Fam105a*	1.54
AU023762	-1.19	*Grin2a*	-1.53
Gh	-1.19	*A_52_P348256*	-1.52
Sln	1.13	*Egr3*	-1.49
A_52_P36261	1.10	*Thbs4*	-1.49
Myh1	1.10	*Spink8*	-1.47
Folr1	1.06	*9430091E24Rik*	-1.43
Sult1c2	0.99	*Wipf3*	-1.42
Tmem72	0.93	*Fabp1*	-1.39
A_51_P417257	-0.93	*Mup5*	-1.38
Chi3l4	-0.89	*2310002F09Rik*	-1.36
Tns1	-0.89	*Tnfrsf25*	-1.36
Cd38	0.88	*Kcne2*	-1.33
Myh4	0.88	*Itpka*	-1.33
Defb22	-0.87	*Hormad1*	-1.32
Mpl	-0.84	*Egr4*	-1.30
Eif2a	-0.83	*Dusp18*	1.24

Table S2: Top 30 fold change probes for each genotype compared to WT in the hypothalamus during the ultradian light-dark cycle.
